# Music and Brain Health: Recommendations From the Global Council on Brain Health, an AARP Collaborative

**DOI:** 10.1093/geroni/igaa057.2835

**Published:** 2020-12-16

**Authors:** Sarah Lock

## Abstract

Music is a complex auditory stimulus that resonates on a physiological, psychological, and spiritual level for people around the world. This symposium will provide highlights from the Global Council on Brain Health consensus report aimed at helping the public to understand the potential that music holds for supporting and enriching brain health. The Global Council on Brain Health (GCBH) is an independent collaborative of scientists, clinicians, scholars, and policy experts convened by AARP to provide evidence-based advice on what people and professionals can do to maintain and improve brain health. The Council translates scientific research into actionable recommendations for the public that will help drive behavior change in individuals across communities and cultures. Issue specialists from around the world were brought together to build consensus, issue recommendations, and offer practical tips. Moreover, we will feature research from our issue experts and provide an overview of the impact of music participation on older adults, including those with dementia. Data from surveys fielded by AARP research, developed in consultation with the GCBH, will also be featured. In sum, this presentation will highlight the work of the Council at the forefront of this international effort to translate advancements in brain health research to the wider public, with an emphasis on individuals aged50 and older.

